# Supplementary material for: Tailoring exciton and excimer emission in an exfoliated ultrathin 2D metal-organic framework
Source: Nat Commun. 2018 Jun 19;9:2401. doi: 10.1038/s41467-018-04833-1 (PMC6008449; doi:10.1038/s41467-018-04833-1)
Supplement: Supplementary file 1 — Supplementary Information [file 41467_2018_4833_MOESM1_ESM.pdf]

Tailoring Exciton and Excimer Emission in an Exfoliated Ultrathin 2D Metal–Organic Framework

Liao et al.

Supplementary Figures

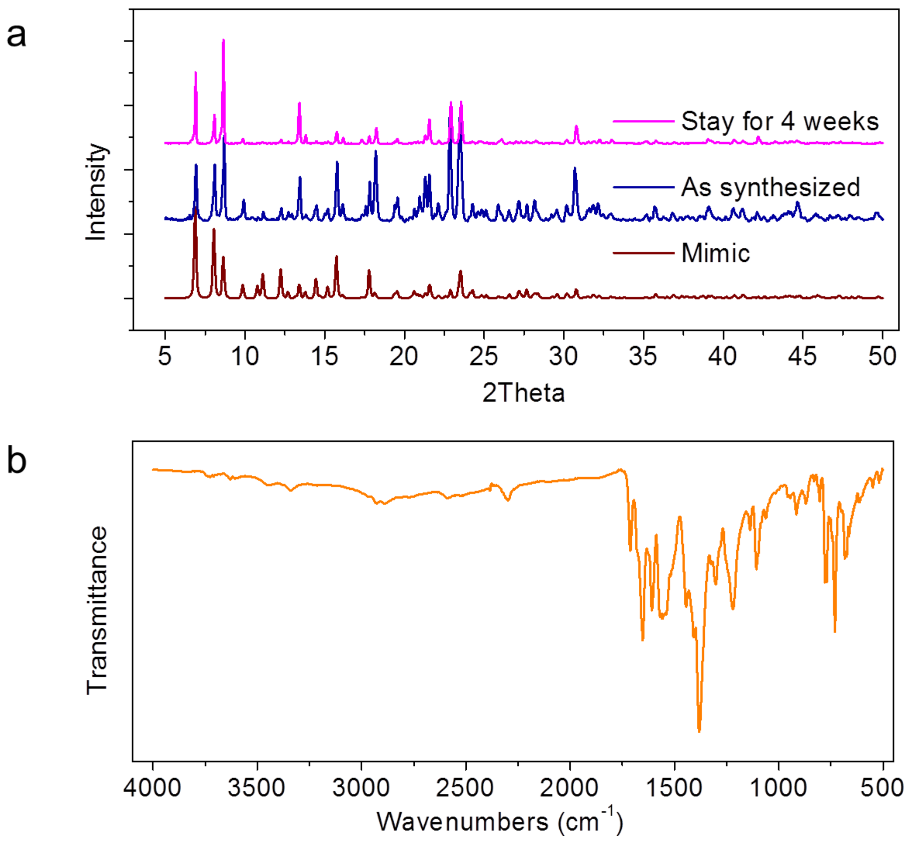

Supplementary Figure 1 | PXRD patterns of as-synthesized and simulated (a), and FT-IR spectrum (b) of Ca-MOF.

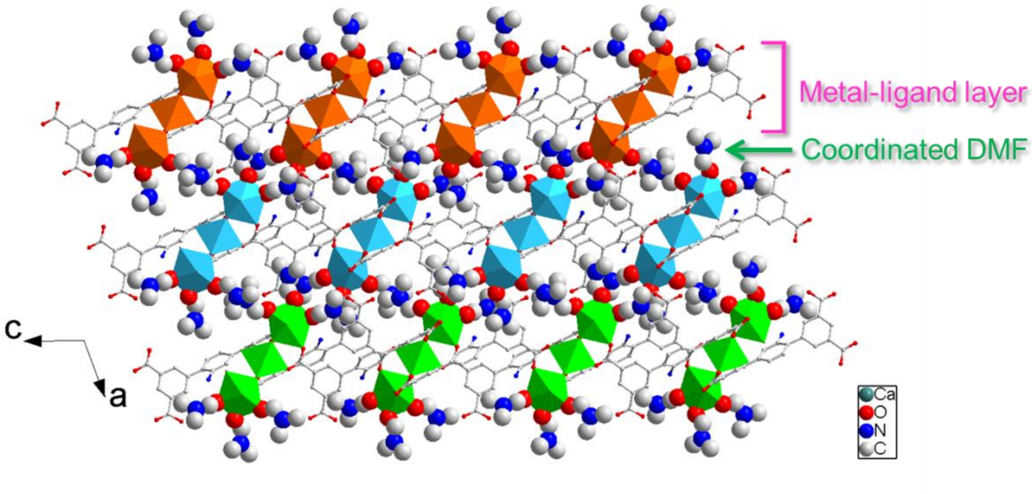

Supplementary Figure 2 | Crystal structure of 2D metal-ligand layers segmented by coordinated DMF molecules in Ca-MOF.

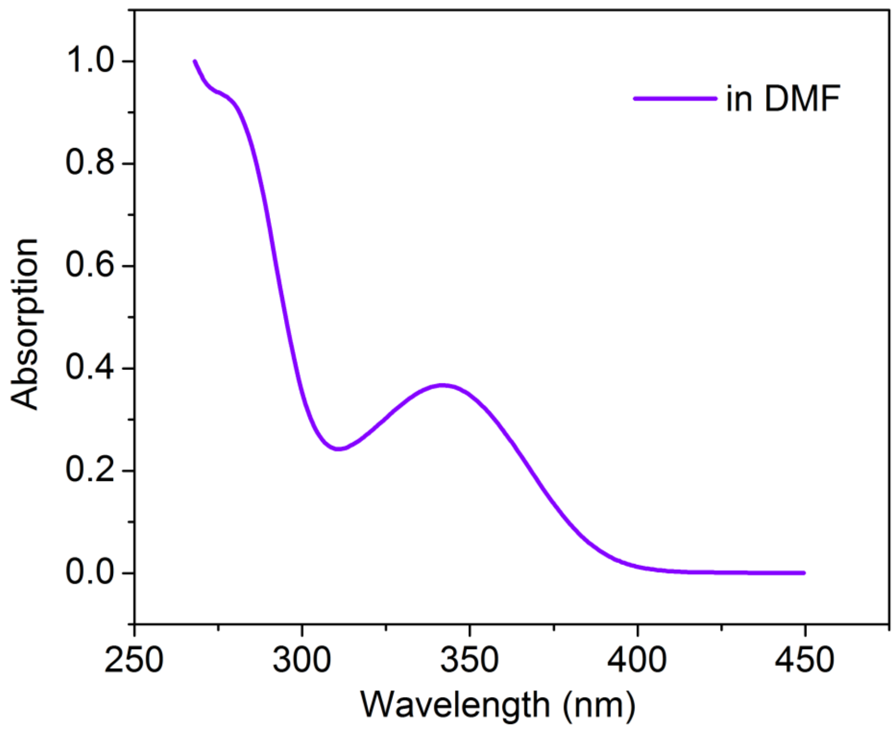

Supplementary Figure 3 | UV-vis absorption spectrum of H_4L ligand in DMF solvent (1×10^{-5} mol l^{-1}).

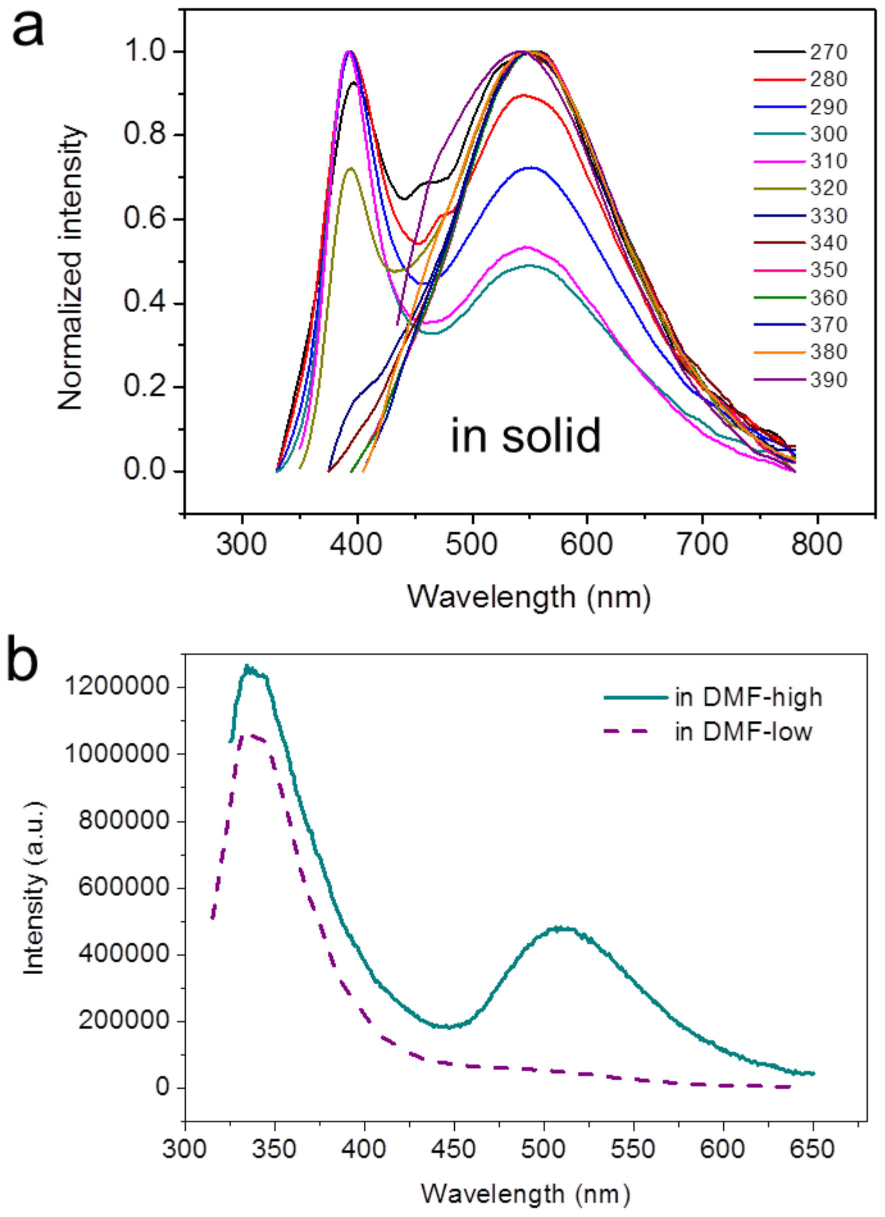

Supplementary Figure 4 | Emission spectra of H_4L ligand in solid at different excitation wavelengths (a), and in low and high concentration of DMF solvent (b, λ_{ex} = 275 nm).

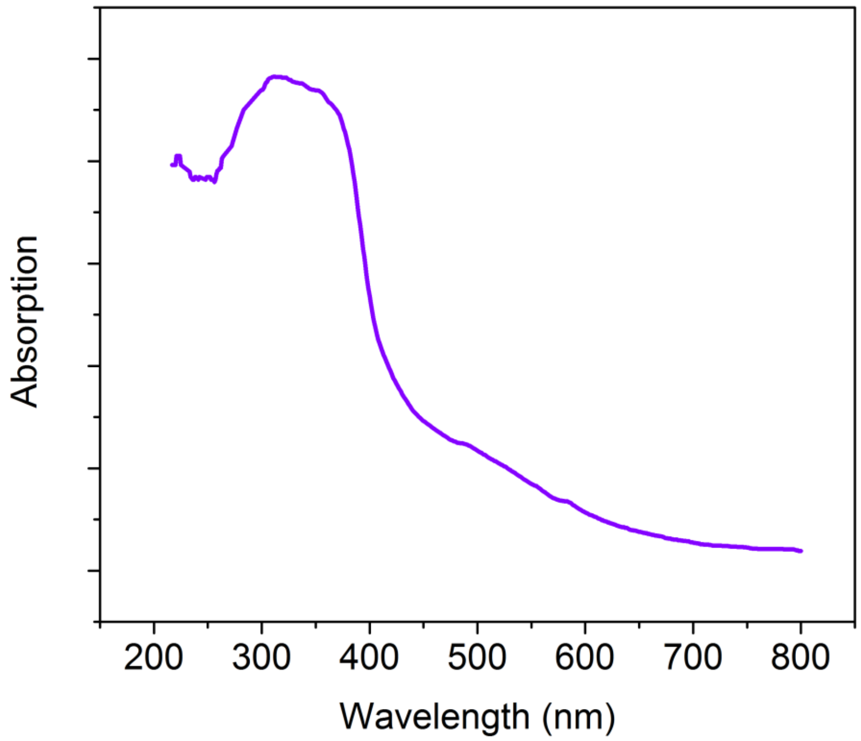

Supplementary Figure 5 | UV-vis absorption spectrum of Ca-MOF bulk crystal in solid state.

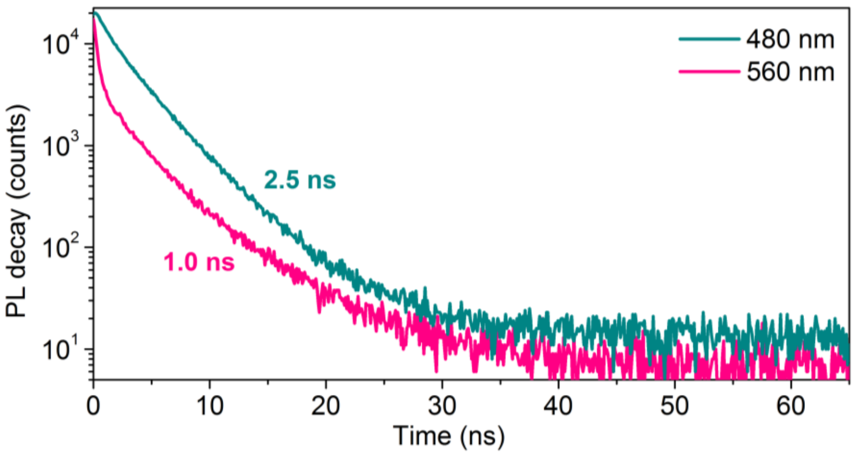

Supplementary Figure 6 | Time decay of interlayer exciton and intralayer ligand-based excimer at 480 and 560 nm.

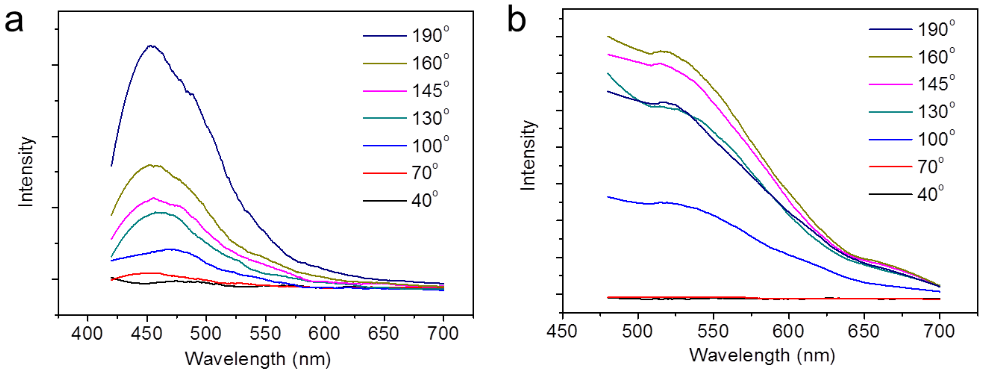

Supplementary Figure 7| Light-polarization-dependent PL spectra of Ca-MOF crystal with different polarization angle excited at 405 (a) and 460 nm (b).

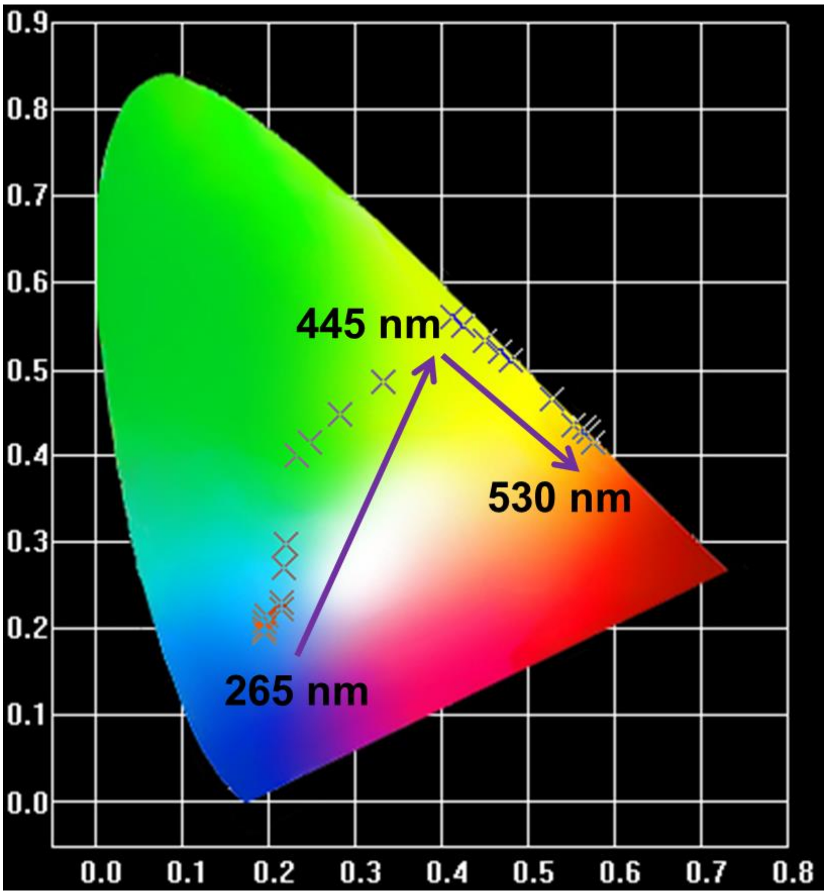

Supplementary Figure 8 | CIE coordinates of the emission spectra of Ca-MOF bulk crystal with different excitation wavelengths.

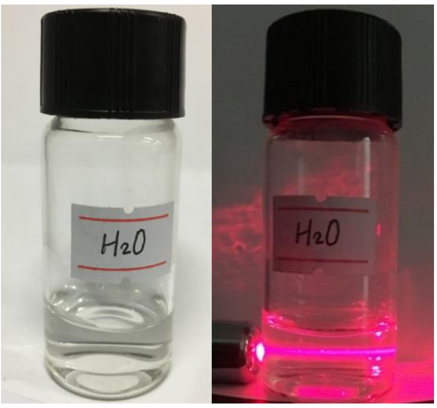

Supplementary Figure 9│ Tyndall effect through the supernatant after Ca-MOF was sonicated in water without (left) and with (right) a laser pointer.

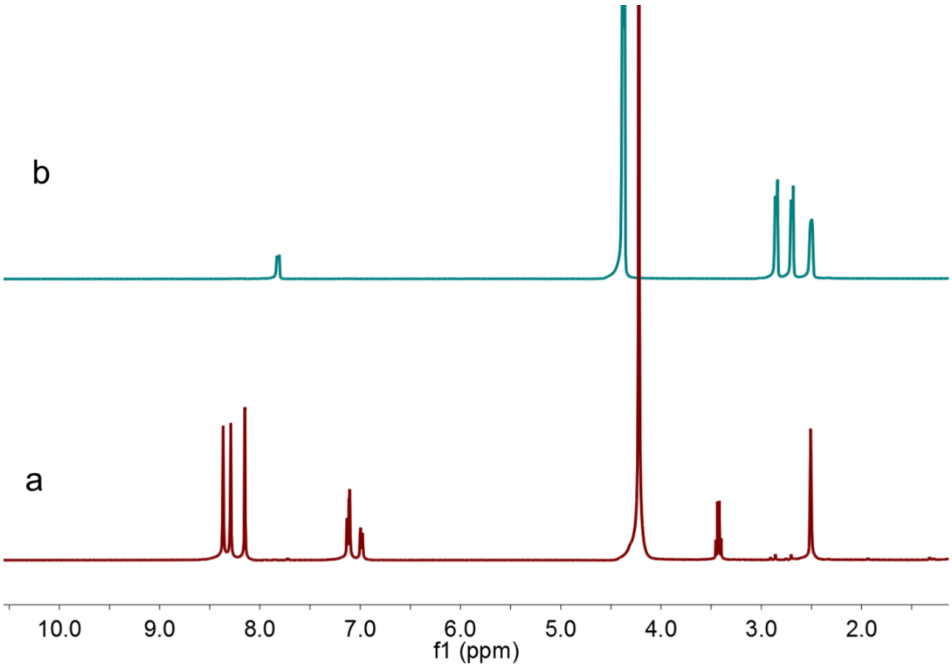

Supplementary Figure 10 | ^{1}H NMR spectra of H_4L ligand (a) and the filtrate after Ca-MOF exfoliation.

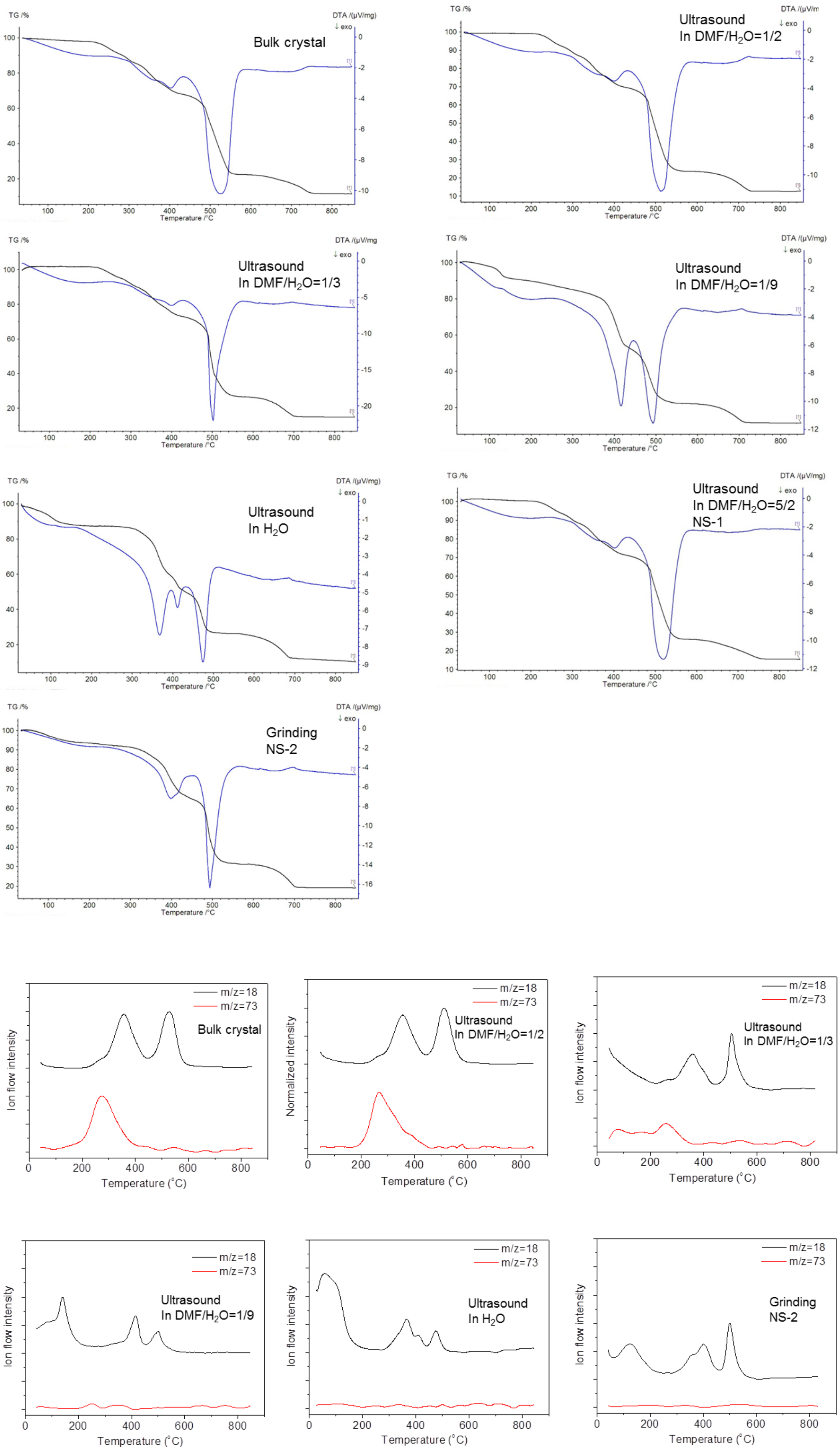

Supplementary Figure 11 | TG-MS spectra of different morphological samples of Ca-MOF.

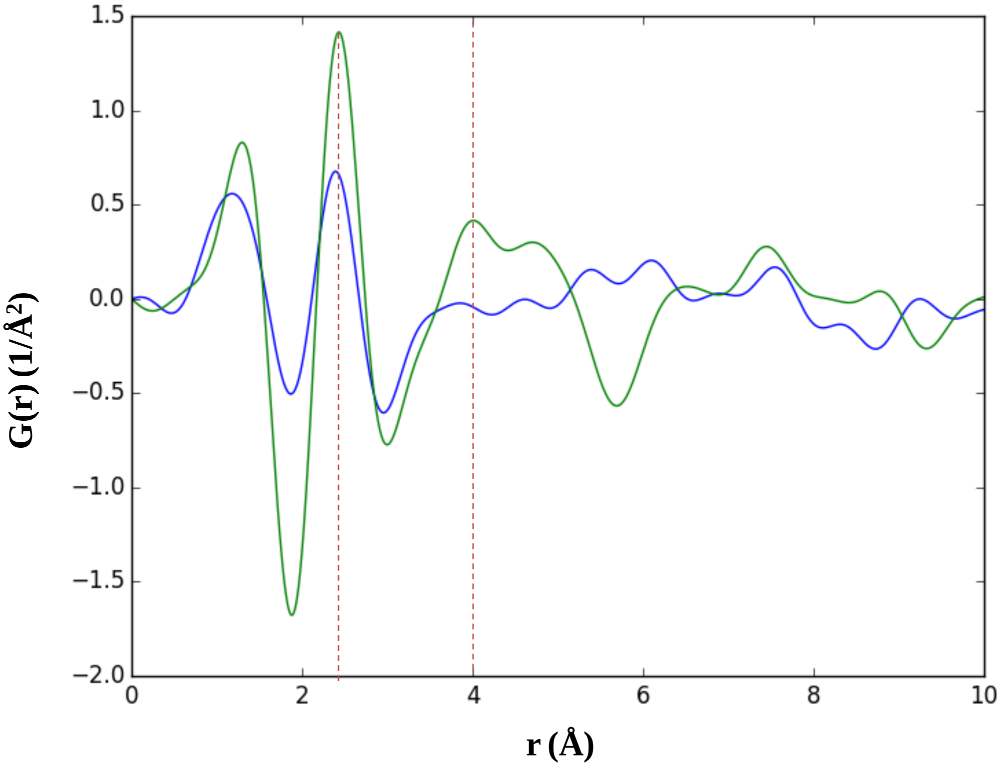

Supplementary Figure 12 | PDF pattens of the nanobelt (green) and bulk crystal (blue) samples of Ca-MOF. G(r) is the experiment obtained reduced pair distribution function, $G(r) = 4\pi r\rho_0(g(r) - 1)$, in which $g(r)$ is the pair distribution function, ρ_0 is the average number density. More details concerning the definition and derivation of PDF technique in case of nano materials can be found in the literature[1].

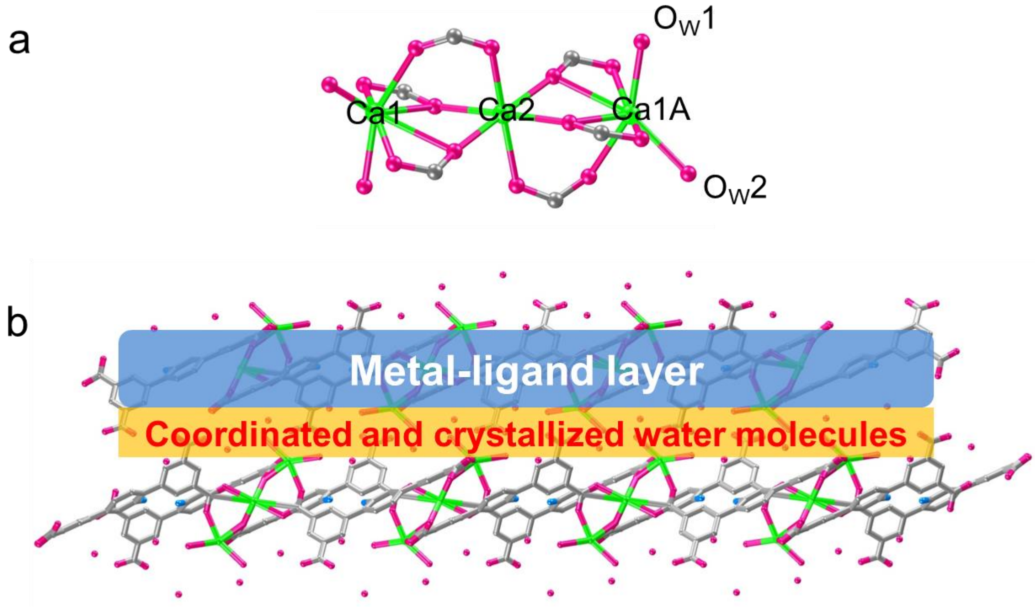

Supplementary Figure 13 | Crystal structure of 2D Ca-MOF-H_2O: {Ca_3O_{16}} metal-oxygen cluster (a) and view of 2D metal-ligand coordination layers along *b* direction (b). Atom color: Calcium, green; Carbon, gray; Oxygen, pink; Nitrogen, blue; Hydrogen atoms are omitted for clarity.

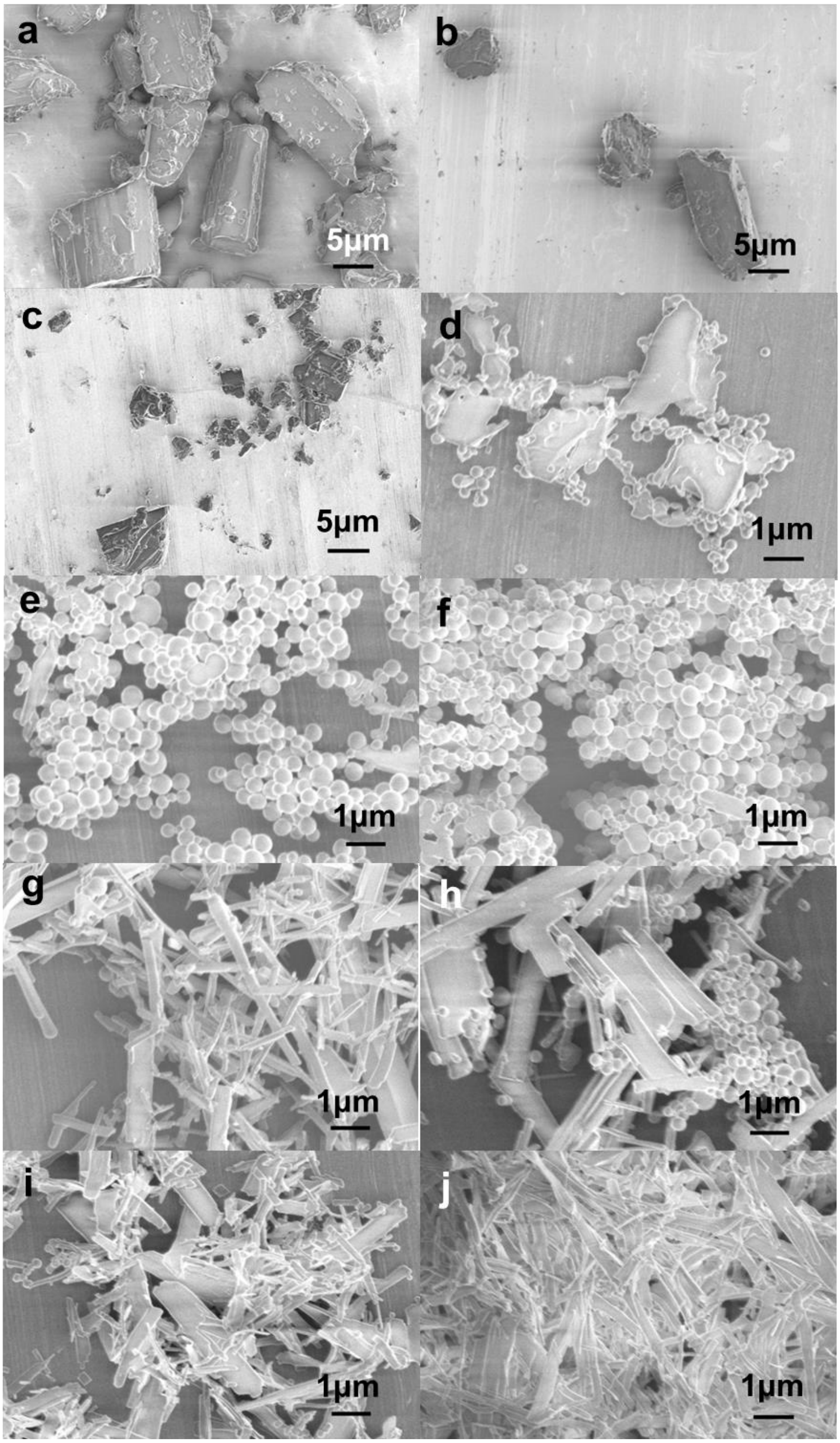

Supplementary Figure 14 | SEM images of Ca-MOF after ultrasound in different water content of DMF/H_2O mixture. (a) 20%, (b) 29%, (c) 33%, (d) 50%, (e) 67%, (f) 75%, (g) 80%, (h) 85%, (i) 90%, (j) 95%.

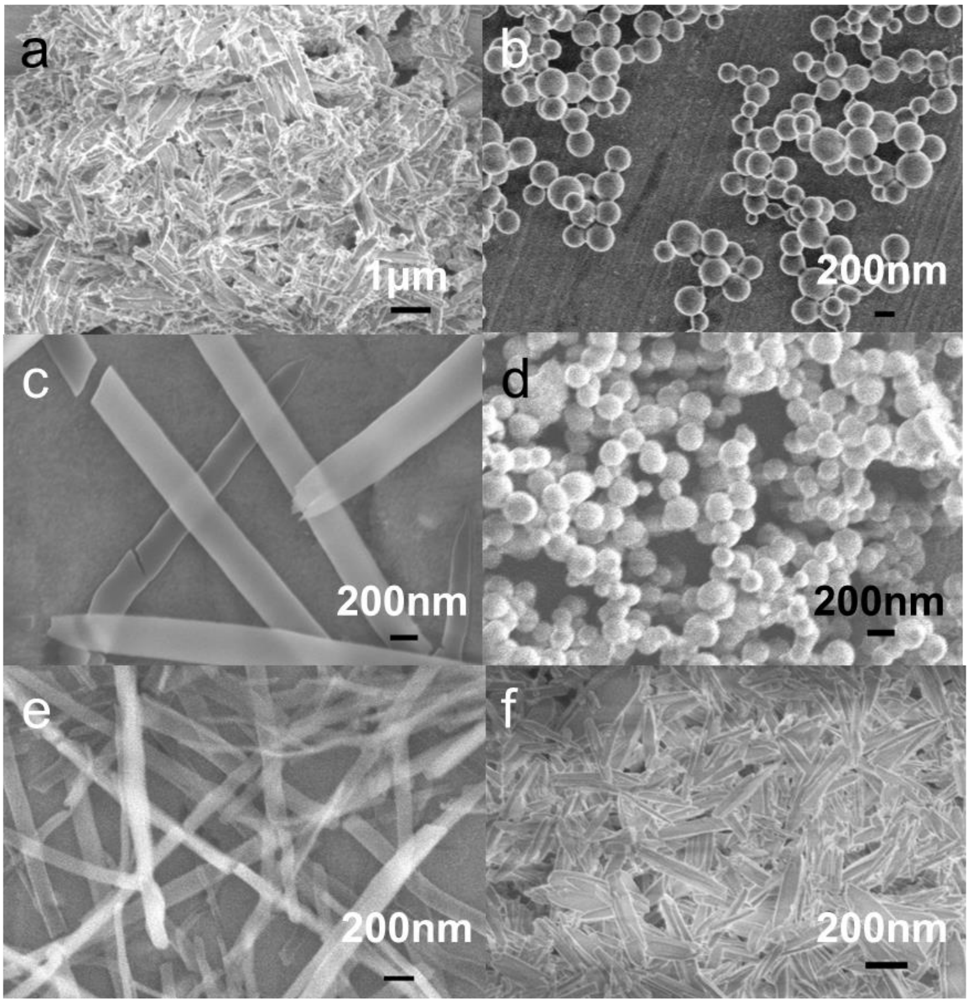

Supplementary Figure 15|SEM images of mutual transformations of nanobelts, nanospheres and nanosheets by ultrasound in different solvents: nanobelts to NS-1 (a) and nanospheres (b), NS-1 to nanobelts (c) and nanospheres (d), nanospheres to nanobelts (e) and NS-1 (f).

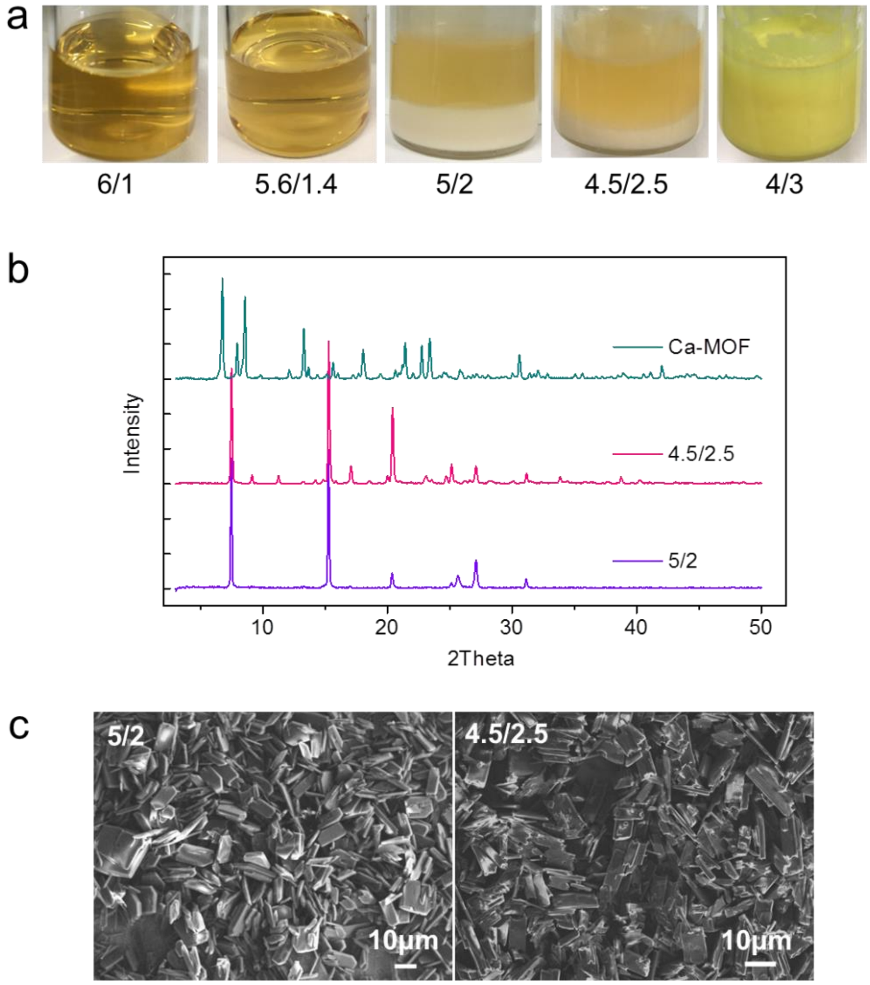

Supplementary Figure 16 | (a) The mixtures obtained after ultrasonicating the concentrate solution of the precursors with various ratios of DMF/H_2O. (b) PXRD and (c) SEM images of precipitates obtained from ultrasonicating the concentrate solution of the precursors with ratios of DMF/H_2O 5/2, and 4.5/2.5, respectively.

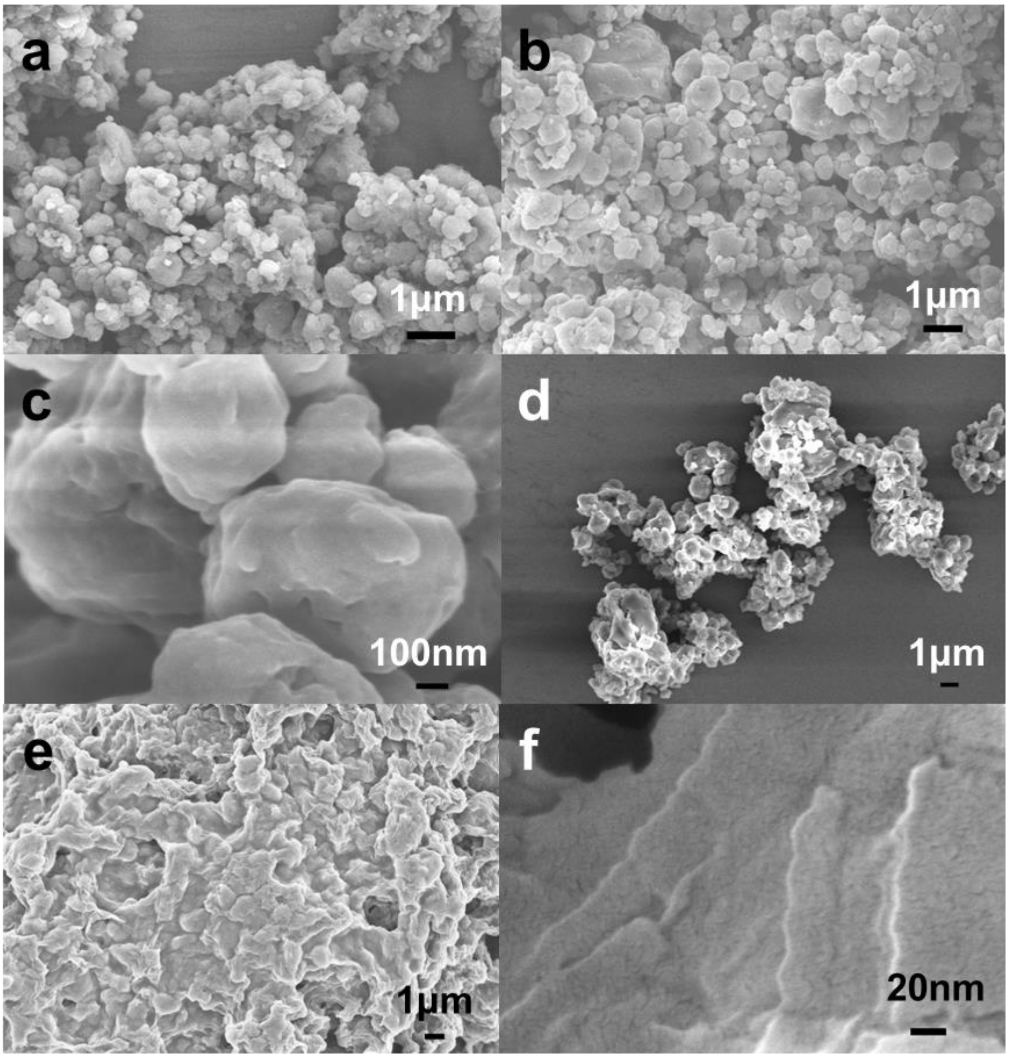

Supplementary Figure 17 | SEM images of the samples after grinding Ca-MOF bulk crystals for 5 min (a), 8 min (b, c) and 40 min (d) at RH=55%, and after 5 min (e) and 8 min (f) at RH=75%.

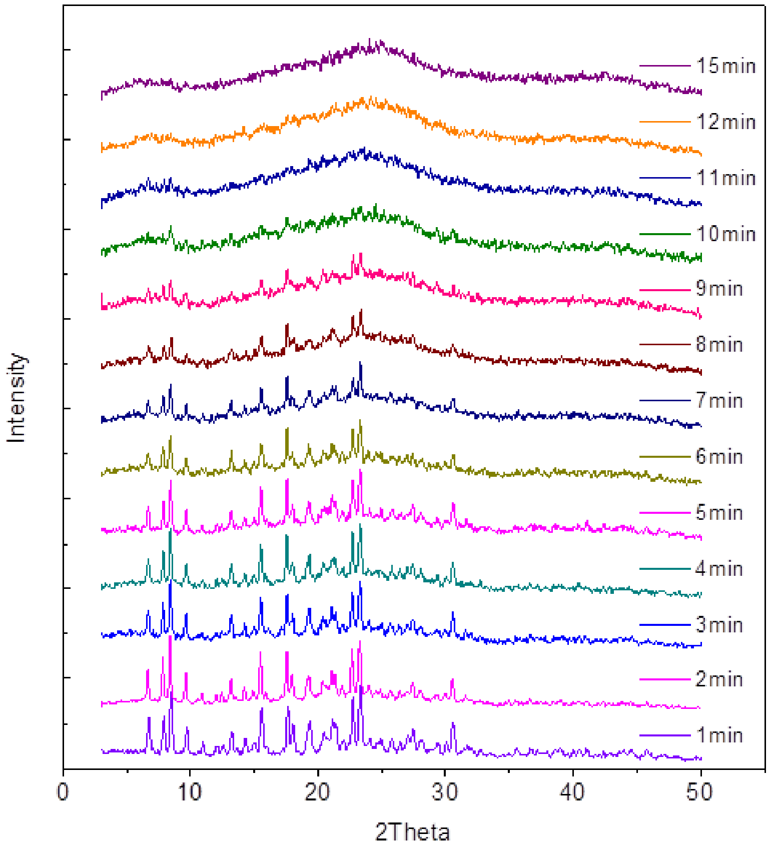

Supplementary Figure 18 | PXRD patterns of Ca-MOF samples after different grinding time at RH=55%.

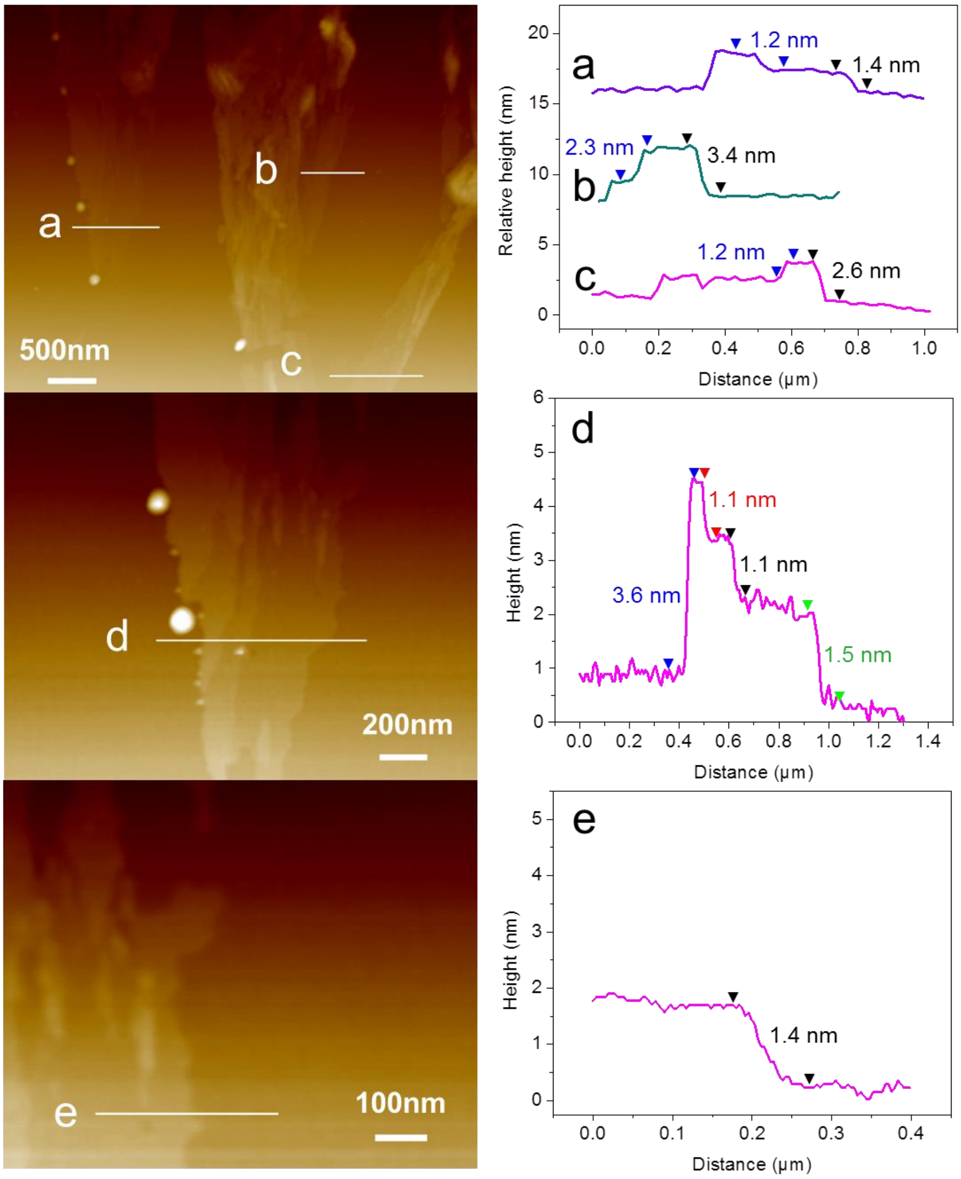

Supplementary Figure 19 | AFM images of the nanosheet (NS-2) samples of Ca-MOF after grinding 8 min at RH=75%.

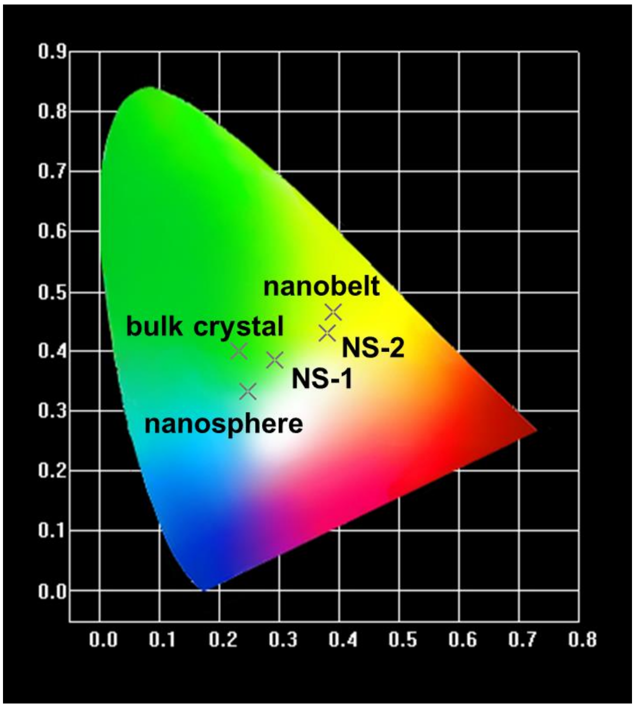

Supplementary Figure 20 | Emitting CIE coordinates of different morphological samples of Ca-MOF.

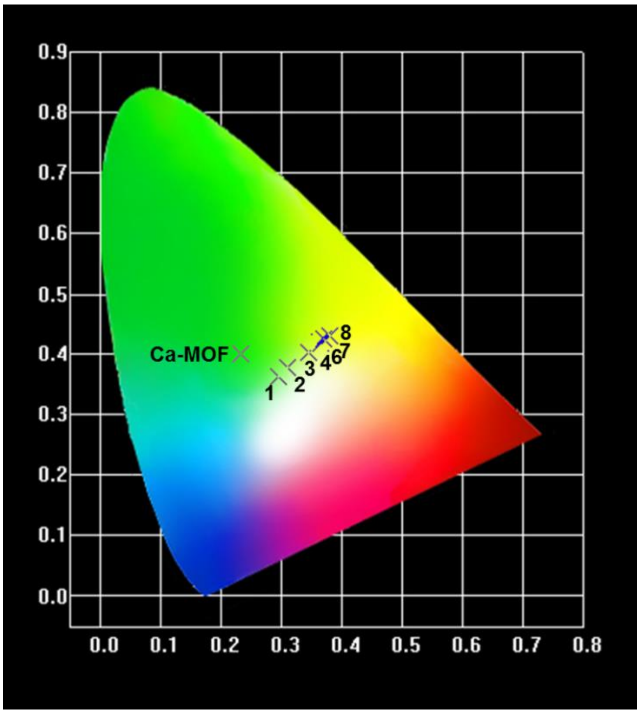

Supplementary Figure 21 | Emitting CIE coordinates of Ca-MOF samples after grinding for different time (1~8 min, respectively).

Supplementary Tables

Supplementary Table 1 | Crystal data and structure refinement for Ca-MOF complex.

Complex	Ca-MOF	Ca-MOF-H_2O
Temperature	150 K	150 K
Chemical formula	$C_{59}H_{59}N_7O_{21}Ca_3$	$C_{44}H_{40}Ca_3N_2O_{24}$
CCDC number	1584130	1823260
Formula weight	1322.37	1101.02
crystal system	Monocline	Monocline
space group	*C2/c*	*P2/n*
a (Å)	23.3335(8)	15.772(2)
b (Å)	9.9658(3)	9.9539(10)
c (Å)	27.2952(7)	17.157(3)
α (deg)	90	90
β (deg)	109.773(4)	108.852(17)
γ (deg)	90	90
V (Å^3)	5972.9(3)	2549.1(7)
Z	4	2
D_{Calcd} (g cm^{-3})	1.471	1.434
μ (mm^{-1})	3.137	3.572
Ref. collected	9333	8237
Independent ref.	4940	4420
R_{int}	0.0320	0.0767
Goodness of fit	1.027	1.057
$R1^a$ [$I > 2\sigma(I)$]	0.0463	0.1171
wR_2^b [$I > 2\sigma(I)$]	0.1335	0.2860

^{a} $R_1=\Sigma(||F_0|- |F_c||)/\Sigma|F_0|$; ^{b} $wR_2=[\Sigma w(F_0^2- F_c^2)^2/\Sigma w(F_0^2)^2]^{1/2}$

Supplementary Table 2 | Luminescence lifetimes of Ca-MOF bulk crystals, nanospheres, nanobelts, and nanosheets (NS-1 and NS-2) detected at the maximum emission.

	bulk crystal	nanosphere	nanobelt	NS-1	NS-2
Lifetime (ns)	2.5	1.7	0.8	1.5	1.2

Supplementary Table 3 | Composition analyses of different Ca-MOF samples.

	Composition	Preparation	EA (tested)/%			EA (calculated)/%		
			C	H	N	C	H	N
1	$[Ca_3(HL)_2(DMF)_5]_n$ (bulk crystal)		53.12	4.54	7.28	53.59	4.46	7.41
2	$[Ca_3(HL)_2(DMF)_{4.5}(H_2O)_{0.5}]_n$ (nanosphere)	Ultrasound in DMF/H_2O(1:2)	53.63	4.49	7.10	53.31	4.37	7.03
3	$[Ca_3(HL)_2(DMF)_4(H_2O)]_n$ (nanosphere)	Ultrasound in DMF/H_2O(1:3)	52.99	4.26	6.69	53.05	4.26	6.63
4	$\{[Ca_3(HL)_2(DMF)(H_2O)_4]\cdot 4H_2O\}_n$ (mixed nanosphere and nanobelt)	Ultrasound in DMF/H_2O(1:9)	48.48	4.21	3.32	48.05	4.00	3.58
5	$\{[Ca_3(HL)_2(H_2O)_5]\cdot 4H_2O\}_n$ (nanobelt)	Ultrasound in H_2O	47.50	3.78	2.50	47.20	3.75	2.50
6	$[Ca_3(HL)_2(DMF)_{4.5}(H_2O)_{0.5}]_n$ (NS-1)	Ultrasound in DMF/H_2O(5:2)	52.84	4.52	7.17	53.31	4.37	7.03
7	$\{[Ca_3(HL)_2(DMF)_{0.5}(H_2O)_{4.5}]\cdot 5H_2O\}_n$ (NS-2)	grinding	47.28	3.95	2.65	46.93	3.99	3.01

Supplementary Methods

The X-ray diffraction data of Ca-MOF was collected on an Agilent Technologies SuperNova system equipped with Cu-Kα radiation (λ = 1.54178 Å). The crystal was kept at 150(2) K during data collection process. The structure was solved with the ShelXS structure solution program integrated in Olex2 using Direct Methods, and refined with the ShelXL refinement package.[2] For Ca-MOF, one benzene group of ligand was disordered, so it was split into two parts for refinement. Meanwhile, one DMF molecule was disordered and refined by splitting into three parts. SIMU and SADI constraints were applied to the disordered benzene ring and DMF molecule. For Ca-MOF-H_2O, OMIT was used to delete several bad data points. We have tried to refine with these data points, but some unreasonable bonds appeared. All non-hydrogen atoms of these two crystals were treated anisotropically. The positions of the hydrogen atoms are generated geometrically. The refinement result of Ca-MOF and Ca-MOF-H_2O were as following.

In order to prove the ligand didn't decompose during the exfoliation process, D_2O was used for ultrasound solvent. In detail, 10 mg Ca-MOF was added into 2 mL D_2O and the mixture was sonicated for 20 min. 2 mL d_6-DMSO was used to wash the solid after ultrasound. The supernatant was separated by centrifuge and filtered with 0.2 μm filter membranes for three times (to exclude the effect of nanoparticles). The obtained filtrate was conducted for ^{1}H NMR and compared with H_4L ligand. It showed that only the peak of DMF molecule (7.95, 2.89 and 2.73 ppm) was found in the filtrate. No other ligand-based peak was observed between 6.8 and 8.5 ppm. This result could prove that Ca-MOF did not decompose to produce ligand during the exfoliation process in water.

Supplementary References

1 Farrow C. L. & Billinge, S. J. L. Relationship between the atomic pair distribution function and small-angle scattering: implications for modeling of nanoparticles. *Acta Crystallogra. A* **65**, 232 (2009).

2 Dolomanov, O. V.; Bourhis, L. J.; Gildea, R. J.; Howard, J. A. K. & Puschmann, H. OLEX2: a complete structure solution, refinement and analysis program. J. *Appl. Crystallogr.* **42**, 339 (2009).
