# Supplementary material for: Tailoring exciton and excimer emission in an exfoliated ultrathin 2D metal-organic framework
Source: Nat Commun. 2018 Jun 19;9:2401. doi: 10.1038/s41467-018-04833-1 (PMC6008449; doi:10.1038/s41467-018-04833-1)
Supplement: Supplementary file 3 — Description of Additional Supplementary Files [file 41467_2018_4833_MOESM3_ESM.pdf]

Description of Additional Supplementary Files

File Name: Supplementary Data 1
Description: Single crystal data of Ca-MOF.

File Name: Supplementary Data 2
Description: Single crystal data of Ca-MOF-H2O.
